# Metformin Beyond Diabetes: A Precision Gerotherapeutic and Immunometabolic Adjuvant for Aging and Cancer

**DOI:** 10.3390/cancers17152466

**Published:** 2025-07-25

**Authors:** Abdul Rehman, Shakta Mani Satyam, Mohamed El-Tanani, Sainath Prabhakar, Rashmi Kumari, Prakashchandra Shetty, Sara S. N. Mohammed, Zaina Nafees, Basma Alomar

**Affiliations:** 1 Department of Pathology, RAK College of Medical Sciences, RAK Medical and Health Sciences University, Ras Al Khaimah 11172, United Arab Emirates; 2 Department of Pharmacology, RAK College of Medical Sciences, RAK Medical and Health Sciences University, Ras Al Khaimah 11172, United Arab Emirates; 3 RAK College of Pharmacy, Ras Al Khaimah Medical and Health Sciences University, Ras Al Khaimah 11172, United Arab Emirates; 4 Department of Perfusion Technology, Manipal College of Health Professions, Manipal Academy of Higher Education, Manipal 576104, India; 5 Department of Operations, Kasturba Hospital, Manipal Academy of Higher Education, Manipal 576104, India; 6 Department of Anatomy, Manipal University College Malaysia, Melaka 75150, Malaysia

**Keywords:** repurposing, biguanide, anti-diabetic, anti-cancer, anti-aging, senolytics

## Abstract

Metformin, a long-established antidiabetic agent, is undergoing a renaissance as a prototype gerotherapeutic and immunometabolic oncology adjuvant. Mechanistic advances reveal that metformin modulates an integrated network of metabolic, immunological, microbiome-mediated, and epigenetic pathways that impact the hallmarks of aging and cancer biology. Clinical data now demonstrate its ability to reduce cancer incidence, enhance immunotherapy outcomes, delay multimorbidity, and reverse biological age markers. Landmark trials such as UKPDS, CAMERA, and the ongoing TAME study illustrate its broad clinical impact on metabolic health, cardiovascular risk, and age-related disease trajectories. In oncology, trials such as MA.32 and METTEN evaluate its influence on progression-free survival and tumor response, highlighting its evolving role in cancer therapy. This review critically synthesizes the molecular underpinnings of metformin’s polypharmacology, examines results from pivotal clinical trials, and compares its effectiveness with emerging gerotherapeutics and senolytics. We explore future directions, including optimized dosing, biomarker-driven personalization, rational combination therapies, and regulatory pathways, to expand indications for aging and oncology. Metformin stands poised to play a pivotal role in precision strategies that target the shared roots of aging and cancer, offering scalable global benefits across health systems.

## Simple Summary

Metformin, originally developed for type 2 diabetes, is now being recognized for its broader potential in promoting healthy aging and enhancing cancer treatment. It affects several core biological pathways involved in aging, cancer progression, and immune regulation. These include metabolic processes, inflammation, gut microbiota composition, and gene expression. By acting on these interconnected systems, metformin may help reduce cancer risk, boost the effectiveness of immunotherapy, and delay the onset of multiple age-related conditions. It is also linked to signs of slower biological aging. This review explores the molecular mechanisms behind metformin’s wide-ranging actions, examines evidence from clinical studies, and considers how it compares with emerging anti-aging treatments. Future directions include tailoring metformin use through biomarker-based personalization and combining it with other therapies for greater benefit. As an affordable and well-tolerated medication, metformin holds significant promise as a versatile tool for extending healthspan and improving outcomes in aging and cancer care.

## 1. Introduction

Metformin developed from its initial discovery as an herbal compound in *Galega officinalis* through the years into a primary therapeutic treatment for diabetes [1]. The discovery of guanidine in 1918 and the synthesis of dimethyl biguanide in 1922 served as essential steps [2]. Jean Sterne’s clinical work during the 1950s validated the medication leading to Glucophage’s approval as a glucose-lowering agent [3]. Metformin existed as a medicine for many years before scientists discovered its ability to activate AMPK and inhibit hepatic gluconeogenesis [4].

In diabetic patients, the risk of colorectal and liver cancers more than doubled without treatment, but low-dose metformin (≤500 mg/day) significantly lowered cancer incidence to close to non-diabetic levels—showing especially strong reductions in colorectal cancer for women and liver cancer for men (adjusted hazard ratios: colorectal 0.36, liver 0.06) [5]. The significant epidemiological discovery of metformin’s multiple actions across metabolic processes and immune responses and epigenetic effects and microbial alterations caused a surge in scientific studies about its pleiotropic effects.

During the last twenty years, metformin has evolved into a multi-targeted therapeutic agent. Preclinical studies have established the potential of metformin as a cancer-preventive agent [6], and randomized clinical trials have demonstrated its ability to prevent the development of precancerous lesions [7]. However, clinical trials assessing its efficacy in treating established cancers have so far yielded disappointing results [8]. Two primary hypotheses have been proposed to explain metformin’s potential antitumor effects: (i) a direct mechanism, where metformin inhibits mitochondrial function in malignant or premalignant cells, and (ii) an indirect mechanism, where it lowers hepatic gluconeogenesis—thereby reducing circulating glucose, insulin, and IGF-1 levels [9,10]. Metformin, through AMPK activation during metabolic stress, induces phosphorylation of histone H2B at serine 36 (H2BS36ph), leading to chromatin remodeling that reactivates tumor suppressor genes such as p21, thereby contributing to its anticancer effects by promoting cell cycle arrest and inhibiting tumor progression [11].

Metformin enhances the oxidative phosphorylation and energy metabolism of CAR-T cells, promoting their proliferation and activation. When co-delivered via a metformin-loaded alginate hydrogel scaffold implanted at the tumor site, metformin not only suppresses cancer cell metabolism and reduces tumor hypoxia but also induces CAR-T cells to adopt a long-lived, highly activated phenotype. This synergistic approach significantly improves CAR-T cell infiltration and antitumor efficacy against both local and distant tumors, while minimizing systemic immune-related side effects, offering a promising strategy to enhance CAR-T therapy for solid tumors using clinically approved drugs and biomaterials [12]. The TAME trial is investigating metformin’s ability to delay the onset of multiple age-related chronic diseases by targeting the fundamental biology of aging. In this randomized, placebo-controlled study of 3000 older adults (65–79 years) without diabetes, the participants receive metformin (1500 mg/day) or a placebo over four years. The primary outcome is a reduction in the incidence of major age-related conditions, including cardiovascular disease, cancer, cognitive decline, and mortality, with 90% power to detect a 22.5% risk reduction. Secondary outcomes assess physical and cognitive function, as well as common geriatric syndromes. The trial also establishes a biorepository to identify the biomarkers and mechanisms through which metformin exerts its geroprotective effects, shaping regulatory pathways for future aging-targeted therapies [13].

As our understanding of the biological mechanisms underlying aging and cancer continues to expand, it has become increasingly evident that many chronic diseases share common pathways including dysregulated metabolism, chronic inflammation, immune senescence, genomic instability, and epigenetic drift. Metformin, originally developed as an antidiabetic agent, has now emerged as a promising gerotherapeutic and immunometabolic modulator that targets these fundamental processes. Mechanistic studies reveal that metformin acts through a polypharmacological network, influencing mitochondrial function, AMPK signaling, epigenetic remodeling, gut microbiota composition, and immune cell metabolism [14,15,16,17]. Metformin can reduce cancer incidence, enhance the efficacy of immunotherapies (such as CAR-T cells), slow multimorbidity progression, and lower biological age as measured by validated epigenetic clocks [18,19,20,21].

Despite these advances, significant knowledge gaps persist. The precise mechanisms by which metformin exerts its geroprotective and anticancer effects remain incompletely defined, particularly regarding tissue-specific actions, dose-response relationships, and interaction with the tumor microenvironment. Furthermore, robust biomarkers that predict individual responsiveness to metformin, as well as optimal combination strategies with other gerotherapeutics (e.g., rapalogs, senolytics) or cancer immunotherapies, have yet to be established. Regulatory frameworks for aging-targeted interventions are also evolving, with landmark trials like TAME helping to shape the path toward formal indications for aging-related conditions.

In this context, the present review aims to provide a comprehensive and critical synthesis of the molecular underpinnings of metformin’s gerotherapeutic and immunometabolic effects. We examine key findings from pivotal clinical trials, compare metformin’s efficacy to emerging geroscience interventions, and explore the potential of biomarker-driven precision medicine approaches. Additionally, we highlight opportunities for rational combination therapies and discuss regulatory and translational pathways to accelerate metformin’s integration into aging and oncology care. Ultimately, this review seeks to inform the future positioning of metformin as a scalable, globally accessible strategy to modulate the biology of aging and cancer in clinical practice.

## 2. Methodology

A comprehensive literature review was conducted to explore the off-label potential of metformin as a precision gerotherapeutic and immunometabolic adjuvant in aging and cancer. A systematic search across PubMed and Scopus was performed for studies published in the last 10 years, using Boolean operators to combine keywords as follows: (“metformin” AND “aging”) OR (“metformin” AND “cancer”) OR (“metformin” AND “repurposing”) OR (“metformin” AND “gerotherapeutic”) OR (“metformin” AND “immunometabolic”). Inclusion criteria focused on experimental and translational studies involving non-diabetic populations, with articles required to be in English and have full-text availability. Select review articles were included when they provided mechanistic depth or summarized large-scale clinical data. Data extraction followed a standardized template capturing key variables such as study design, population characteristics, metformin dosage, primary outcomes, and safety data. Commentary pieces, abstracts lacking full data, and non-peer-reviewed sources were excluded. This methodology ensured a rigorous and focused synthesis of the emerging role of metformin beyond its traditional antidiabetic use.

## 3. Molecular Pharmacology: Systems Level Mechanisms

Metformin’s therapeutic versatility stems from its multifaceted actions across various biological systems, beginning at the fundamental level of mitochondria where its partial inhibition of Complex I elevates the AMP/ATP ratio, activating AMPK and inducing metabolic stress in tumor cells while paradoxically stabilizing mitochondrial function to protect cardiomyocytes from chemotherapy [22,23]. This AMPK activation extends systemically, influencing the liver by inactivating CRTC2 and downregulating gluconeogenic enzymes like PEPCK and G6Pase, thereby improving glycemic control by reducing hepatic glucose output, and within skeletal muscle, where it drives GLUT4 translocation and PGC1 alpha-mediated mitochondrial biogenesis to enhance glucose uptake and rejuvenate mitochondrial capacity [24,25,26]. Beyond these metabolic effects, metformin offers direct cardiovascular protection by stabilizing mitochondrial membrane potential in cardiomyocytes against doxorubicin-induced cardiotoxicity; it profoundly modulates the gut microbiome by expanding *Akkermansia muciniphila* and influencing FXR/TGR5, leading to enhanced gut barrier integrity and systemic anti-inflammatory effects [27,28,29,30]. Furthermore, metformin induces beneficial epigenetic remodeling via AMPK-driven phosphorylation, opening chromatin at tumor suppressor loci and reversing senescence-associated gene silencing, while crucially reprogramming the tumor microenvironment by reducing lactate and HIF-1 alpha, repolarizing M2 macrophages to M1, and increasing T-cell infiltration to enhance immune cell recruitment and checkpoint blockade responses [31,32]. Finally, its systemic immunomodulation through NRF2 activation, reduction in senescence-associated secretory phenotype (SASP) factors and NLRP3 inflammasome activity, and enhanced antiviral T-cell responses collectively delay immunosenescence, reduce inflammaging, and bolster overall immune surveillance (Table 1) [33,34,35].

These pleiotropic actions extend beyond glycemic control, encompassing mitochondrial modulation, epigenetic remodeling, tumor microenvironment regulation, and immunometabolic reprogramming. The outlined effects help in explaining metformin’s potential as a gerotherapeutic and anticancer agent.

Metformin modulates a broad spectrum of effects which start with mitochondrial complexes before extending to systemic physiology through multiple interconnected mechanisms. Metformin attaches to the ND3 subunit of complex I which results in partial proton pumping inhibition (by ~67%) that boosts the AMP/ATP ratio and activates AMPK. The liver uses CRTC2 inactivation together with decreased PEPCK (−72%) and G6Pase (−68%) expression to inhibit gluconeogenesis. Muscle tissue benefits from AMPK activation, which results in GLUT4 translocation (+58%) to improve insulin sensitivity and glucose absorption [47,48,49,50].

Metformin induces minimal mitochondrial oxidative stress (ROS ↑1.8-fold), which activates NRF2 and antioxidant defense mechanisms similarly to exercise or caloric restriction [51]. When metformin binds to VDAC 1 at higher concentrations, it stabilizes the mitochondrial permeability transition pore to protect the heart while creating selective toxicity in cancer cells [36,52]. Through the inhibition of FXR and the activation of TGR5, metformin causes a substantial change in the gut microbiome, while *Akkermansia muciniphila* grows substantially to enhance gut barrier integrity and minimize endotoxemia and boost GLP 1 secretion [53,54,55]. The local effects produced by metformin explain why it demonstrates significant gastrointestinal actions together with systemic immunological effects.

Metformin works epigenetically to create H2BS36ph which enables chromatin structure changes that promote tumor suppressor gene enrichment [56,57,58]. The process of aging decreases when epigenetic markers near FOXO3 and SIRT1 show lower values, while circRNA_0001805 expression decreases and senescence-associated circRNAs undergo downregulation [33,59,60,61]. These epigenetic transformations establish a theoretical framework connecting cell metabolic pathways with stress responses and aging mechanisms. The polypharmacological properties of metformin link together metabolic systems with microbial networks and epigenetic mechanisms. The effective utilization of metformin through precision medicine frameworks requires advanced multi-omics and computational systems biology techniques to break down its interrelated pathways.

## 4. Metabolic Reprogramming and Immunomodulation

**Figure 1:**
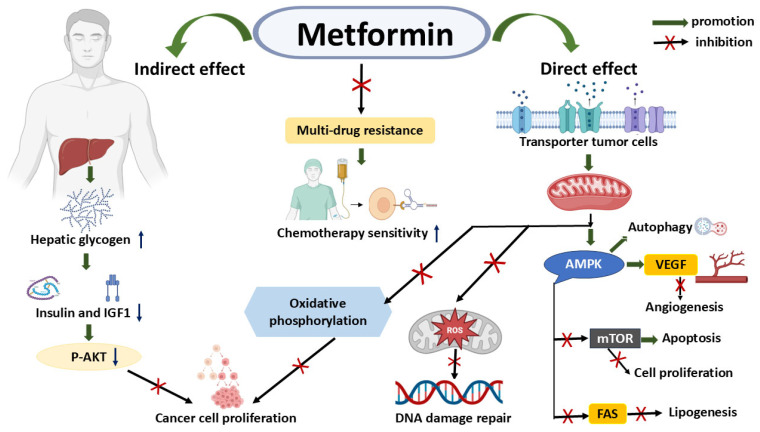
Metformin’s direct and indirect anticancer mechanisms. Abbreviations: AMPK (Adenosine Monophosphate-activated Protein Kinase), FAS (Fatty Acid Synthase), IGF-1 (Insulin-like Growth Factor 1), mTOR (mammalian Target of Rapamycin), P-AKT (Phosphorylated AKT, referring to protein kinase B), ROS (Reactive Oxygen Species), and VEGF (Vascular Endothelial Growth Factor).

Metformin exerts its anticancer effects through both systemic metabolic modulation and direct actions within the tumor microenvironment. Indirectly, it enhances hepatic glycogen synthesis, which leads to a reduction in circulating insulin and insulin-like growth factor 1 (IGF-1) [62]. This decrease in growth-promoting signals results in the suppression of AKT phosphorylation (P-AKT), thereby limiting downstream signaling pathways responsible for uncontrolled cancer cell proliferation [63]. Simultaneously, metformin acts directly within tumor cells by entering through specific transporters and targeting essential cellular functions. It inhibits mitochondrial respiration and oxidative phosphorylation, thereby disrupting the cancer cells’ energy production [36,64]. Additionally, it reduces the accumulation of harmful reactive oxygen species (ROS) and impairs DNA damage repair pathways, increasing the susceptibility of cancer cells to stress [65]. One of the key mechanisms involves the activation of AMP-activated protein kinase (AMPK), a central energy sensor. Once activated, AMPK promotes autophagy, suppresses angiogenesis by inhibiting vascular endothelial growth factor (VEGF), and blocks the mTOR signaling pathway, a crucial driver of cell growth and proliferation [66,67]. Furthermore, AMPK downregulates fatty acid synthase (FAS), thereby reducing lipogenesis, a metabolic process frequently upregulated in cancer (Figure 1) [4,68]. These combined actions not only inhibit cancer cell survival and proliferation but also sensitize tumors to chemotherapy and help overcome multidrug resistance, making metformin a valuable complementary agent in oncologic therapy.

This figure was created using the BioRender Basic Plan (BioRender Web Application, available at: https://app.biorender.com/, accessed on 27 June 2025). Images were captured using Snipping Tool (Microsoft, Snipping Tool version 11.2505.21.0), and assembled using Microsoft PowerPoint (Microsoft Office Home and Student 2021) to produce the final composite figure. In Figure 1, straight/curved/linked arrows (→) indicate activation or promotion, arrows with a cross above it (→⊗) indicate blocked or inhibited activation of a process or pathway, upward arrows (↑) represent increase or upregulation, and downward arrows (↓) represent decrease or downregulation. This figure comprehensively illustrates the distinct and overlapping mechanisms through which metformin exerts its anticancer effects, categorized into indirect and direct pathways. Indirect effects primarily involve systemic metabolic changes: Metformin increases hepatic glycogen synthesis, leading to a decrease in circulating insulin and insulin-like growth factor 1 (IGF-1) levels. Reduced insulin and IGF-1 signaling subsequently inhibits the phosphorylation of AKT (P-AKT), a key pro-survival and proliferative kinase. This inhibition ultimately suppresses cancer cell proliferation. Additionally, metformin indirectly contributes to improved chemotherapy sensitivity by mitigating multidrug resistance, possibly through its systemic metabolic effects. Direct effects on tumor cells are mediated by metformin’s uptake via specific transporters. Within tumor cells, metformin critically impacts mitochondrial function. It activates adenosine monophosphate-activated protein kinase (AMPK), a central energy sensor. AMPK activation leads to multiple anticancer outcomes: it inhibits mammalian target of rapamycin (mTOR), thereby decreasing cell proliferation and promoting apoptosis; it reduces vascular endothelial growth factor (VEGF) expression, which inhibits angiogenesis; and it suppresses fatty acid synthase (FAS), thereby limiting lipogenesis essential for cancer cell growth. Furthermore, metformin directly reduces reactive oxygen species (ROS) and enhances DNA damage repair. It also inhibits oxidative phosphorylation, further contributing to the suppression of cancer cell proliferation. These direct mechanisms collectively enhance the cytotoxic effects of chemotherapy and combat multidrug resistance.

Metformin exerts profound immunomodulatory effects that enhance antitumor immune responses and synergize with multiple cancer therapies. It plays a key role in shaping a favorable tumor microenvironment by promoting the differentiation of conventional T cells into effector memory T cells, which are critical for the production of the antitumor cytokine interferon-gamma (IFN-γ) [46,69]. At the same time, it suppresses immunosuppressive components of the immune system, such as regulatory T cells (Tregs), and inhibits both the migration and function of myeloid-derived suppressor cells (MDSCs), which typically blunt immune activation in tumors [70,71]. Metformin has emerged as a promising immunometabolic modulator within the tumor microenvironment (TME), exerting pleiotropic effects on both innate and adaptive immune responses. A well-documented mechanism involves its ability to reprogram tumor-associated macrophages (TAMs), shifting them from an anti-inflammatory, immunosuppressive M2 phenotype toward a pro-inflammatory, antitumorigenic M1 phenotype, thereby reinstating their ability to secrete cytokines such as IL-12 and TNF-α, enhancing antigen presentation and promoting cytotoxic T cell recruitment [72]. In parallel, accumulating evidence demonstrates that metformin significantly enhances the effector functions of natural killer (NK) cells by modulating their metabolic and immune checkpoint profiles. Metformin has been shown to restore mitochondrial integrity, increase oxidative phosphorylation, and reduce glycolytic overdependence in NK cells exposed to the hypoxic and glucose-depleted TME, thereby rescuing their metabolic fitness and longevity [73,74,75]. Furthermore, metformin downregulates the expression of inhibitory checkpoint receptors such as PD-1 and TIM-3 on dysfunctional NK cells, attenuating TME-induced exhaustion and reinvigorating their cytolytic activity [44]. This immunorestorative effect is corroborated by increased expression of CD107a, a degranulation marker reflecting NK cell-mediated cytotoxicity, as well as elevated secretion of IFN-γ and granzyme B [74,76]. Moreover, metformin facilitates NK cell trafficking into tumor tissue by upregulating CXCR3 expression and remodeling chemokine gradients through inhibition of HIF-1α and lactate accumulation [77,78,79,80]. Collectively, these mechanistic insights underscore metformin’s role in enhancing innate immune surveillance, reducing immune exhaustion, and reprogramming the immunosuppressive TME, thereby potentiating its therapeutic synergy when used as an adjuvant in combination with checkpoint inhibitors or other cytotoxic agents in oncology.

**Figure 2:**
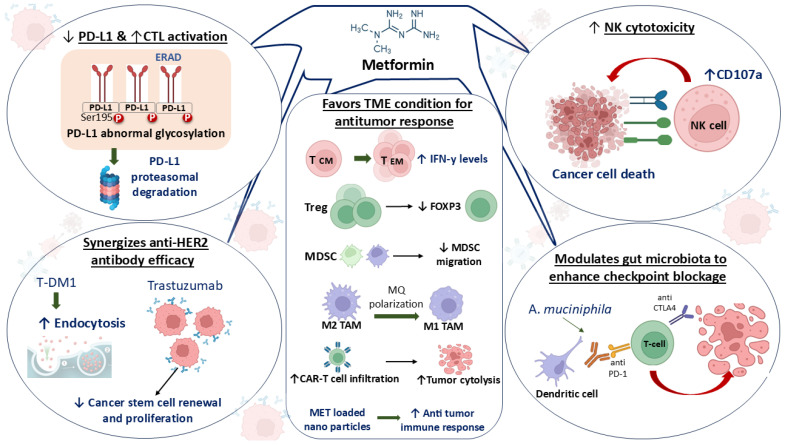
Metformin shapes antitumor immunity and enhances immunotherapy. Abbreviations: *A. muciniphila (Akkermansia muciniphila)*, CAR T (Chimeric Antigen Receptor T-cell), CD107a (Cluster of Differentiation 107a, also known as LAMP-1, a marker for degranulation in NK cells), CTL (Cytotoxic T Lymphocyte), CTLA4 (Cytotoxic T-Lymphocyte-Associated protein 4), ERAD (Endoplasmic Reticulum-Associated Degradation), FOXP3 (Forkhead box protein P3), HER2 (Human Epidermal growth factor Receptor 2), IFN-γ (Interferon-gamma), MDSC (Myeloid-Derived Suppressor Cells), MET (Metformin, when referring to loaded nanoparticles), MQ (Macrophage), NK cell (Natural Killer cell), PD-1 (Programmed Death-1), PD-L1 (Programmed Death-Ligand 1), P-PD-L1 Ser195 (Phosphorylated Programmed Death-Ligand 1 at Serine 195), Tcm (Central Memory T cell), Tem (Effector Memory T cell), TME (Tumor Microenvironment), T-DM1 (Trastuzumab emtansine, an antibody-drug conjugate targeting HER2), and Treg (Regulatory T cell).

Another critical mechanism involves its regulation of immune checkpoint pathways; metformin promotes proteasomal degradation of PD-L1, likely through modulation of its aberrant glycosylation at Ser195 via the ER-associated degradation (ERAD) pathway [76,81]. This results in reduced PD-L1 expression on tumor cells, effectively releasing inhibitory signals on cytotoxic T lymphocytes (CTLs) and enhancing their tumor-killing potential [82]. Metformin also exhibits synergy with targeted therapies, including anti-HER2 agents such as trastuzumab and T-DM1, by increasing T-DM1 internalization, thus boosting its efficacy against cancer stem cells [83]. Moreover, it influences the gut microbiota, particularly by enriching beneficial species like *Akkermansia muciniphila*, which further potentiates the effectiveness of immune checkpoint inhibitors targeting PD-1 and CTLA-4 (Figure 2) [84,85,86]. This microbial-driven immunopotentiation ultimately contributes to stronger T-cell activation and improved elimination of cancer cells.

This figure was created using the BioRender Basic Plan (BioRender Web Application, available at: https://app.biorender.com/, accessed on 27 June 2025). Images were captured using Snipping Tool (Microsoft, Snipping Tool version 11.2505.21.0), and assembled using Microsoft PowerPoint (Microsoft Office Home and Student 2021) to produce the final composite figure. In Figure 2, straight/curved/linked arrows (→) indicate activation or promotion, upward arrows (↑) indicate increased expression or activation and downward arrows (↓) indicate decreased expression or inhibition. This figure illustrates how metformin actively remodels the tumor microenvironment (TME) to enhance antitumor immunity and synergize with various cancer immunotherapies. Metformin creates a TME favorable for immune activation by modulating key immune cell populations: it promotes the differentiation and expansion of central memory T cells (Tcm) and effector memory T cells (Tem), both critical for durable immune responses and associated with elevated IFN-γ levels; it reduces the number and suppressive function of regulatory T cells (Treg) by downregulating FOXP3 expression; and it diminishes the migration and immunosuppressive activity of myeloid-derived suppressor cells (MDSCs). Additionally, metformin shifts macrophage polarization from pro-tumorigenic M2-like tumor-associated macrophages (TAMs) to antitumorigenic M1-like TAMs. It also enhances CAR T-cell infiltration into tumors, increasing tumor cell lysis, and when delivered via nanoparticles (MET-loaded nanoparticles), it further amplifies the immune response. Metformin exhibits distinct synergistic effects with immunotherapies by downregulating PD-L1 through ERAD-mediated aberrant glycosylation and proteasomal degradation (via Ser195 phosphorylation), thereby improving cytotoxic T lymphocyte (CTL) activation. It boosts natural killer (NK) cell cytotoxicity by upregulating CD107a expression and enhances the efficacy of anti-HER2 therapies (e.g., trastuzumab and T-DM1) by promoting antibody endocytosis and suppressing cancer stem cell renewal. Furthermore, metformin modulates the gut microbiota, increasing the abundance of *Akkermansia muciniphila*, which contributes to improved responses to immune checkpoint inhibitors (e.g., anti-CTLA4 and anti-PD-1) by supporting T-cell activation and tumor destruction.

The cancer-vulnerability targeting properties of metformin arise from its ability to interfere with metabolism as well as alter tumor microenvironments and boost the immune system. Metformin disrupts tumor bioenergetics by targeting mitochondrial complex I thus triggering AMPK activation and mTORC1 suppression [87]. PI3K/AKT/mTOR pathway dysregulation makes cancer cells particularly vulnerable to this approach, leading to substantial reductions in protein synthesis and proliferation rates [88]. Reduced oxygen usage reduces HIF 1α instability and VEGF levels and increases radio sensitivity [89,90]. Metformin activates MHC I expression and activates dendritic cells and changes M2 macrophages into M1 cells [45]. Metformin together with pembrolizumab in NSCLC patients leads to improved ORR rates from 28% to 49% and shows the best outcomes in lactate-rich tumor environments [91,92]. Studies reported that metformin acts against cancer stem cells by reducing pluripotency factor expression while lowering ALDH+ cell numbers and reducing tumor-initiating potential by 90% [93,94,95].

## 5. Addressing the Hallmarks of Aging

Metformin exerts geroprotective effects by modulating multiple hallmarks of aging, notably through the suppression of cellular senescence and the restoration of mitochondrial function. Its activation of AMPK signaling leads to a marked reduction in key senescence markers, including SA-β-galactosidase and p16^INK4a—a cyclin-dependent kinase inhibitor encoded by the CDKN2A gene [96,97]. Additionally, metformin significantly attenuates the release of senescence-associated secretory phenotype (SASP) factors, reducing IL-6 levels by 58% and MMP-3 by 64%, thereby mitigating the pro-inflammatory environment associated with aging [61,98,99]. The ATM-p53 pathway enhances DNA repair processes, and the PINK1-Parkin pathway increases mitophagy [100].

**Figure 3:**
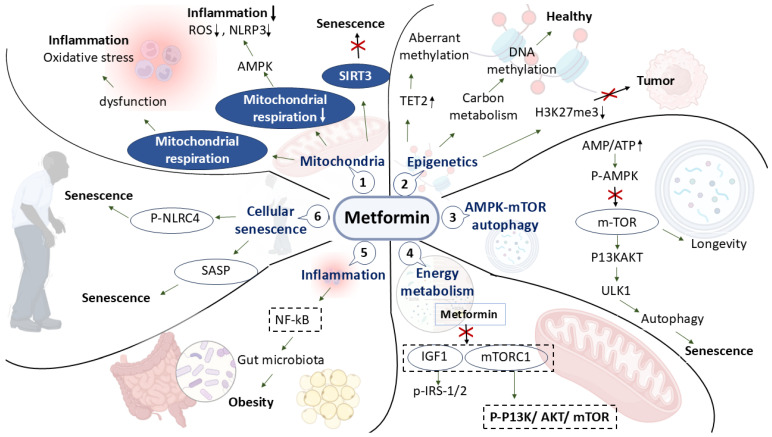
Metformin’s multi-targeted anti-aging and metabolic effects. Abbreviations: AMPK (Adenosine Monophosphate-activated Protein Kinase), AMP/ATP (Adenosine Monophosphate/Adenosine Triphosphate), H3K27me3 (Tri-methylation of Lysine 27 on Histone H3), IGF1 (Insulin-like Growth Factor 1), IRS-1/2 (Insulin Receptor Substrate 1/2), mTOR (mammalian Target of Rapamycin), mTORC1 (mammalian Target of Rapamycin Complex 1), NF-κB (Nuclear Factor kappa-light-chain-enhancer of activated B cells), NLRP3 (NOD-, LRR-, and pyrin domain-containing protein 3), P-AKT (Phosphorylated AKT), P-AMPK (Phosphorylated AMPK), P-NLRC4 (Phosphorylated NLRC4—NLR family CARD domain-containing 4), P-PI3K (Phosphorylated Phosphoinositide 3-kinase), ROS (Reactive Oxygen Species), SASP (Senescence-Associated Secretory Phenotype), SIRT3 (Sirtuin 3), TET2 (Ten-Eleven Translocation 2), and ULK1 (Unc-51-Like Autophagy Activating Kinase 1).

Metformin combats aging and promotes longevity through six interconnected biological pathways. One key mechanism is its direct modulation of mitochondrial function, where it suppresses mitochondrial respiration, leading to a reduction in reactive oxygen species (ROS) and inhibition of the NLRP3 inflammasome [101]. This alleviates oxidative stress and chronic inflammation, both of which are central contributors to age-related cellular damage [33,102,103,104,105,106]. Metformin also influences epigenetic regulation by upregulating TET2 and reducing repressive H3K27me3 histone marks, fostering a gene expression profile associated with genomic stability and youthful cellular function [107,108]. Another major mechanism involves activation of AMP-activated protein kinase (AMPK), which in turn inhibits the mTOR pathway [109]. This promotes autophagy, a critical process that clears damaged proteins and organelles, thereby preserving cellular homeostasis and extending cellular lifespan. Additionally, metformin modulates systemic metabolic signaling by reducing levels of insulin-like growth factor 1 and suppressing mTORC1 activity, both of which are implicated in the acceleration of cellular senescence [33]. Its anti-inflammatory actions further contribute to longevity by inhibiting NF-κB, a key transcription factor in inflammatory responses, and by reshaping the gut microbiota in ways that reduce obesity-related inflammation. Metformin directly attenuates cellular senescence and suppresses the senescence-associated secretory phenotype (SASP) through inhibition of phosphorylated NLRC4 (NOD-like receptor family CARD domain-containing 4), thereby limiting chronic pro-inflammatory signaling and tissue dysfunction associated with aging (Figure 3) [110]. The NLRP3 inflammasome and NF κB signaling pathways of metformin reduce CRP and IL 6 levels [111].

This figure was created using the BioRender Basic Plan (BioRender Web Application, available at: https://app.biorender.com/, accessed on 27 June 2025). Images were captured using Snipping Tool (Microsoft, Snipping Tool version 11.2505.21.0), and assembled using Microsoft PowerPoint (Microsoft Office Home and Student 2021) to produce the final composite figure. In Figure 3, straight/curved/linked arrows (→) indicate activation or promotion, arrows with a cross above it (→⊗) indicate blocked or inhibited activation of a process or pathway, upward arrows (↑) represent increase or upregulation, and downward arrows (↓) represent decrease or downregulation. This figure elucidates the diverse mechanisms by which metformin influences key cellular processes related to aging, metabolism, and inflammation, often contributing to its anti-senescence and health-promoting effects. 1. Mitochondrial Respiration: Metformin inhibits mitochondrial respiration, which in turn reduces oxidative stress and inflammation (indicated by decreased ROS and NLRP3). This inhibition is linked to improved mitochondrial function and the suppression of senescence. 2. Epigenetics: Metformin modulates epigenetic marks, including decreasing aberrant DNA methylation and increasing TET2 activity. It also influences carbon metabolism and reduces H3K27me3, thereby contributing to altered gene expression that may counteract tumor formation. 3. AMPK–mTOR–Autophagy Pathway: Metformin activates AMPK, leading to an increased AMP/ATP ratio and phosphorylation of AMPK (P-AMPK). This activation inhibits mTOR, a crucial pathway regulating cell growth and proliferation. Inhibition of mTOR promotes autophagy (via ULK1), a cellular self-cleaning process vital for longevity and cellular health, while suppressing senescence. 4. Energy Metabolism: Metformin directly affects energy metabolism by inhibiting key pathways involving IGF1 and mTORC1, which regulate insulin signaling (P-IRS-1/2) and downstream PI3K/AKT/mTOR pathways, collectively influencing cellular growth and senescence. 5. Inflammation: Metformin mitigates inflammation by inhibiting NF-κB, a central mediator of inflammatory responses. This anti-inflammatory action also contributes to reducing senescence. 6. Cellular Senescence: Metformin directly or indirectly (via reduced inflammation and P-NLRC4) suppresses cellular senescence, a state of irreversible cell cycle arrest associated with aging and disease. The overall effect on senescence, oxidative stress, and inflammation contributes to improved longevity and a healthier metabolic state, partially influenced by gut microbiota and obesity.

## 6. Future Directions Toward Precision Gerotherapeutics and Oncology

The combination of moderate lifespan extension with metabolic advantages and preserved immunity and low-cost safety features makes metformin superior to rapamycin and senolytics and NAD^+^ boosters and lifestyle interventions [112,113]. Metformin, owing to its affordability and broad accessibility, is best utilized as an adjunct that enhances the efficacy of immunotherapies and mTOR-targeted treatments, rather than as a standalone replacement in oncology care.

A growing body of clinical evidence supports the diverse therapeutic effects of metformin across multiple cancer types and in aging-related interventions. Metformin’s repositioning beyond glycemic control is supported by data from several landmark trials. The UK Prospective Diabetes Study (UKPDS) demonstrated that metformin not only improved glycemic outcomes but also significantly reduced diabetes-related mortality and all-cause mortality in overweight patients with type 2 diabetes [114]. The CAMERA trial further extended metformin’s potential into cardiovascular disease prevention, showing modest reductions in weight and improvement in inflammatory biomarkers in non-diabetic patients with coronary heart disease, although without significant changes in carotid intima-media thickness [115]. The ongoing TAME (Targeting Aging with Metformin) trial represents a paradigm shift, evaluating metformin as a gerotherapeutic agent to delay multimorbidity in aging populations, offering a unique opportunity to test aging biology in a clinical context [116]. In the MA.32 phase 3 randomized, double-blind, placebo-controlled trial involving 3649 non-diabetic patients with high-risk operable breast cancer, adjuvant metformin (850 mg twice daily for 5 years) failed to show a significant improvement in invasive disease-free survival compared to the placebo, with no benefit observed in either hormone receptor-positive or -negative subgroups [117]. The METTEN trial evaluated the addition of metformin (850 mg twice daily) to neoadjuvant chemotherapy and trastuzumab in women with early HER2-positive breast cancer and found a numerically higher pathological complete response (pCR) rate in the metformin arm (65.5%) compared to the control (58.6%), though this difference was not statistically significant (*p* = 0.589), and the regimen was generally well tolerated [118]. Together, these trials underscore metformin’s expanding role from metabolic regulation to aging and cancer therapeutics.

As summarized in Table 2, trials in breast, prostate, ovarian, and hematologic malignancies have demonstrated mixed outcomes, with some studies showing improved clinical response rates, pathological complete response, and reduced recurrence, while others reported no significant improvement in progression-free or overall survival. Notably, metformin’s adjunctive use in diffuse large B-cell lymphoma (DLBCL) significantly enhanced remission and survival outcomes. In breast cancer, particularly in neoadjuvant settings, metformin showed potential as a chemosensitizer by modulating molecular markers such as DR4, DR5, and CD133. In aging research, the ongoing TAME trial aims to evaluate metformin’s capacity to delay age-related diseases, while the MILES study has already revealed metformin-induced transcriptomic shifts linked to mitochondrial function, metabolism, and inflammation. Collectively, these studies underscore the multifaceted role of metformin in both oncologic and geriatric contexts, meriting further exploration in precision therapeutics (Table 2).

This table comprehensively outlines the diverse findings from pivotal clinical trials investigating metformin’s utility beyond diabetes, primarily focusing on its adjunctive role in various cancers and its emerging potential in targeting aging. This table presents selected interventional trials evaluating metformin across various cancer types and aging-related endpoints. It includes details on study populations, interventions, and primary outcomes. The data reflect metformin’s role as both a potential anticancer agent and a gerotherapeutic intervention. Trials vary in cancer subtype, disease stage, combination therapies, and endpoints, offering insights into metformin’s efficacy, safety, and translational significance in oncology and aging research.

### 6.1. Combination Therapies

Future therapeutic approaches will focus on pairing different agents that target distinct aging and cancer pathways. The broad-spectrum activity of metformin across metabolic networks together with immunologic pathways and microbiome networks and epigenetic mechanisms makes it an optimal candidate for combination therapy development in oncology and gerotherapeutics. Research indicates that pairing metformin with senolytic drugs during aging shows promising results in preclinical studies and initial clinical trials [33,133,134]. Research shows that dasatinib in combination with quercetin selectively destroys senescent cells through their elimination process and decreases their pro-inflammatory secretory activities [135]. These agents produce short-lived effects while their elimination of senescent cells creates brief periods of tissue stress. Metformin achieves its effects by activating AMPK and NRF2 signaling pathways which suppress SASP and prevent new senescent cell formation while maintaining tissue homeostasis after senolytic treatment [136,137]. Combination therapy with metformin and senolytics yields greater improvements in frailty scores and reductions in inflammatory markers compared to either treatment alone [33].

Using metformin together with NAD+ precursors, including nicotinamide riboside (NR) or NMN (nicotinamide mononucleotide), presents a promising therapeutic approach [138]. The aging process involves the reduction of NAD+ which deteriorates both mitochondrial operations and DNA maintenance and sirtuin functionality [139]. Metformin activates AMPK and increases NAMPT levels which helps restore NAD+ metabolism, and NR and NMN provide direct NAD+ pool supplementation [140]. Research indicates that the combined use of these medications leads to enhanced mitochondrial development along with improved mitochondrial operation and muscle strength improvements and longer endurance times [141]. Research groups actively investigate the synergistic effects between low-dose rapalogs and metformin [142]. The chronic administration of rapamycin and its related compounds results in impaired glucose homeostasis and weakened immune responses but metformin’s insulin-sensitizing and immune-preserving effects make it suitable to extend the use of rapalogs for lifespan extension [143,144].

Clinical oncologists, oncology researchers, and oncology pharmacists—actively engaged in both cancer research and patient care—are increasingly exploring the integration of metformin-based combination therapies into standard oncological treatment strategies. The application of immune checkpoint inhibitors such as anti-PD-1 and anti-PD-L1 in cancer treatment has revolutionized therapy but produces unsatisfactory responses across numerous cancer types, especially those with suppressed immune environments [145]. The tumor microenvironment transforms when metformin decreases intratumoral lactate and oxygen consumption, which results in the elimination of immunosuppression while allowing T cells to penetrate the tumor [72]. The clinical trials have demonstrated that combining metformin with checkpoint inhibitors leads to better objective response rates while extending progression-free survival, particularly in tumors exhibiting high glycolytic activity or PTEN deficiency [85]. The hostile metabolic conditions inside tumors create obstacles for CAR-T cell therapies, which show promising results [146]. Metformin removes barriers for CAR-T cells to function longer by lowering competition for glucose and improving mitochondrial stability [12]. Research demonstrates that using metformin alongside radiotherapy creates new opportunities for treatment. Metformin enhances radiation therapy through its ability to reduce hypoxia and improve tumor sensitivity to oxidative damage but produces minimal harm to surrounding normal tissue [89].

Metformin serves as a beneficial component when used with glutaminase inhibitors (CB-839) and lactate dehydrogenase inhibitors to target the metabolic differences of cancer cells [147]. The therapeutic strategies work best against tumors which have mutations in LKB1 or PTEN [148]. Researchers are currently studying combination therapies between metformin and PARP inhibitors as well as CDK4/6 inhibitors since metformin interacts with DNA repair pathways and cell cycle mechanisms [149]. The next stage of metformin combination therapy development depends on selecting appropriate regimens through evaluations of tumor biological characteristics and patient-specific factors. The combination of rational design with multi-omics profiling enables scientists to find the optimal synergistic effects that minimize toxic outcomes.

### 6.2. Optimized Dosing and Delivery

The standard diabetes treatment dose of metformin is well-established, but its ideal application in both aging patients and oncology practice remains unclear. The typical daily dosage of 1500–2000 mg in diabetes patients results in steady-state plasma concentrations that range between 1 and 2 mM [150]. Systemic metabolic modulation together with AMPK activation and microbiome effects can be achieved with these intratumoral levels; yet the cancer cells need higher concentrations to achieve optimal therapeutic effects. Research demonstrates that metformin collects inside tumor cells through pH trapping mechanisms and transporters, which enable it to reach intratumoral levels of 5–20 mM in the acidic and hypoxic tumor environment. The delivery of intratumoral concentrations of metformin to systemic levels becomes restricted because of gastrointestinal tolerability and renal clearance [151]. Different optimized dosing schedules are under development to find optimal solutions. A treatment method involves administering 3000 mg twice weekly in intermittent dosages to produce peak plasma concentrations without ongoing gastrointestinal adverse effects [152]. The initial clinical evidence indicates that this strategy works well with patients and successfully delivers required drug amounts to target sites. Research indicates that delivering metformin through nanoparticles presents an appealing method of treatment [153]. Nanoparticles utilize the EPR effect to passively reach tumors and deliver 8–10 times more drug to these locations [154]. Liposomal formulations provide better drug delivery to tumor sites compared to standard formulations, and they also decrease the amount of drug that enters the bloodstream, which helps to prevent unwanted side effects [155]. Research indicates that direct intratumoral metformin delivery provides both high drug concentration levels and safe treatment tolerance and promising therapeutic results in accessible tumors [156].

Among the adverse effects associated with metformin, gastrointestinal intolerance, particularly diarrhea, remains the most common and often dose-limiting side effect. Common adverse events associated with metformin include gastrointestinal disturbances such as diarrhea, nausea, and bloating usually affect approximately 10–25% of patients and can significantly impair medication adherence, especially in individuals with insulin resistance or pre-existing gastrointestinal sensitivity [157]. In oncology patients, who may already present with compromised nutritional status or early signs of cachexia, the risk of dehydration and further weight loss due to persistent diarrhea is a major clinical concern. These effects are dose-dependent and often transient, improving upon slow titration or switching to extended-release formulations. Vitamin B12 deficiency has also been observed with long-term use. A rare but serious complication is lactic acidosis, typically associated with renal impairment or acute illness. To improve tolerability, strategies such as gradual dose titration, switching from immediate-release (IR) to extended-release (XR) formulations, and careful patient selection are essential. These measures are particularly important when considering metformin as an adjunctive therapy in cancer care, where maintaining performance status and quality of life is paramount.

Metformin demonstrates an oral bioavailability of approximately 50–60% with peak plasma concentrations achieved within 2–3 h for IR and 6–8 h for XR formulations [158]. It is not metabolized by the liver but is excreted unchanged in the urine through renal tubular secretion via organic cation transporters (OCTs) and multidrug and toxin extrusion proteins (MATEs). The elimination half-life ranges between 4 and 8 h. Clinically, metformin is available in IR tablets, XR tablets, and oral solution formulations, facilitating titration and adherence based on patient tolerability and pharmacokinetic needs. Age-related changes in pharmacokinetics demand special attention when determining proper doses for aging patients. Older adults require precise adjustment of medication dosages because their kidneys clear drugs more slowly and their bodies absorb them differently through the gastrointestinal system [159]. The variability in gut microbiota among individuals affects how metformin becomes available in the body and what effects it produces systemically [160]. The use of stepped-up dosing starting from 500 mg per day helps both older adults and their caregivers manage treatment tolerance while improving their ability to follow prescribed medication.

Recent chronotherapy research indicates that when metformin is taken at the right time it will maximize its therapeutic effects [161]. The effectiveness of metformin can be enhanced and side effects reduced through timing its administration according to natural biological patterns of AMPK activity and immune response [162]. Metformin administration at night may improve metabolic pathway targeting; yet morning administration might provide better immune response benefits. More comprehensive testing of these hypotheses in clinical trials remains necessary. Pharmacokinetic modeling together with biomarker-driven feedback will enable precision dosing for both aging and cancer patients.

### 6.3. Precision Biomarkers

Reliable biomarkers need identification for predicting metformin’s effectiveness in achieving precision medicine goals. Recent advancements in oncology have led to the identification of several promising predictive biomarkers for treatment response and prognosis. The responsiveness of tumors to metformin treatment depends on PTEN loss along with LKB1 mutations or PI3K/AKT/mTOR pathway activation [88]. Metformin disrupts tumor metabolic pathways through complex I inhibition and AMPK activation when tumors have these specific alterations [87].

The metabolic markers of LDH within tumors at high levels along with elevated lactate levels help predict treatment benefit because metformin decreases tumor glycolytic flux while reducing acidification [163]. The metabolic changes that occur during treatment become accessible through imaging biomarkers, including FDG-PET [164]. The analysis of tumor microenvironment immunophenotyping enables healthcare providers to identify patients who would benefit from combining metformin with immunotherapy. Tumors showing minimal T cell infiltration together with high glycolytic activity could benefit most from this therapeutic method [44].

The use of epigenetic clocks in gerotherapeutics provides an excellent method to determine patient categories and evaluate their response. The biological aging assessment tools DNAmGrimAge and PhenoAge, along with their related metrics enable tracking of treatment-related biological changes [165]. Biological aging markers, including p16INK4a expression in T cells and SASP factors IL-6, GDF15, and MMPs, allow researchers to assess circulating senescence markers. Systemic inflammatory markers CRP and IL-6 provide clinical outcome correlations because they represent inflammaging.

The gut microbiome provides numerous predictive biomarkers for medical use. Metformin glycemic response prediction in patients depends on the initial presence of *Akkermansia muciniphila* together with specific beneficial microbial species in their gut. Microbiome changes that occur throughout therapy play a role in both clinical benefits and their underlying mechanisms.

These biomarkers need continuous monitoring through multi-omics platforms to enable adaptive treatment approaches. Analyzing genomic, transcriptomic, proteomic, metabolomic, and microbiome data through machine learning can help create complete biomarker signatures which direct personalized therapies. These developing technologies will transform metformin from its current standard therapeutic approach into a precise therapeutic tool.

### 6.4. Regulatory and Translational Pathways

Metformin’s integration into routine clinical practice as a gerotherapeutic or oncology adjuvant faces several regulatory challenges that must be strategically addressed. While the U.S. Food and Drug Administration (FDA) and European Medicines Agency (EMA) have demonstrated openness to novel approval pathways—particularly when supported by validated biomarkers and robust endpoints—the formal recognition of aging as a treatable condition remains controversial. The TAME (Targeting Aging with Metformin) trial, led by the American Federation for Aging Research, serves as a landmark regulatory experiment to establish aging as an indication and is being closely watched as a test case for future gerotherapeutic approvals. In oncology, metformin is advancing through standard regulatory pipelines, with trials like MA.32 and METTEN evaluating efficacy as an adjunct to traditional therapy. Although the FDA has not yet approved aging as a standalone indication, frameworks like the 21st Century Cures Act and increased interest in personalized medicine suggest a shift toward accommodating broader disease-modifying indications for repurposed agents. From a global perspective, metformin’s oral availability, affordability, and safety profile make it a compelling candidate for large-scale implementation, particularly in low- and middle-income countries. Its inclusion in the WHO Essential Medicines List for expanded indications would represent a transformative step in democratizing access to aging and cancer interventions worldwide. To achieve this, harmonized international guidelines must support off-label gerotherapeutic use, with emphasis on biomarker-driven patient selection and rigorous long-term safety monitoring. Realizing metformin’s full potential as a precision health tool will require coordinated efforts between academic institutions, regulatory authorities, and global health organizations.

## 7. Conclusions

The transformation of metformin from an antihyperglycemic medication into a precise gerotherapeutic and oncological therapeutic agent marks a significant milestone in translational research. The combination of its metabolic control with its ability to modify immune responses and microbiome and epigenetic regulation makes metformin an ideal candidate to treat aging and cancer shared roots. Research evidence from both laboratory experiments and clinical trials demonstrates that metformin both increases lifespan and decreases cancer occurrence while making new therapies more effective. Metformin’s complete potential will depend on future advancements in rational combination strategies and optimized dosing regimens and biomarker-guided personalization and supportive regulatory frameworks. Emerging evidence suggests that metformin holds promise as a preventive agent against age-related diseases, even in non-diabetic, otherwise healthy individuals. Its ability to modulate key aging hallmarks, such as chronic low-grade inflammation, mitochondrial dysfunction, and genomic instability, supports its potential role in enhancing healthspan. Ongoing trials like the TAME (Targeting Aging with Metformin) trial aim to investigate this hypothesis by evaluating whether metformin can delay the onset of age-related multimorbidity. In our opinion, while definitive clinical evidence is still evolving, it is plausible that metformin could be repositioned as a geroprotective intervention in the future, particularly in individuals at risk for cardiometabolic disorders, neurodegeneration, or cancer. However, long-term safety, dosing strategies, and individual risk–benefit profiles will need to be thoroughly established before routine preventive use is adopted in clinical practice. This review has certain limitations. First, while efforts were made to prioritize original studies, in some mechanistic or emerging areas, reliance on high-quality reviews was necessary due to a paucity of primary data. Second, heterogeneity among RCTs in endpoints, dosages, and populations complicates direct comparisons. Third, the evolving regulatory landscape and biomarker validation limit immediate translation into clinical guidelines. Lastly, publication bias and selective reporting in trials may skew the evidence base. The successful implementation of metformin would establish it as a globally available treatment that effectively delays aging-related diseases and enhances cancer treatment outcomes while serving as a model for modern precision medicine approaches.

## Figures and Tables

**Table 1:** Systemic and molecular targets of metformin.

System/Target	Primary Mechanisms	Functional Outcome
Mitochondria [36]	Partial inhibition of Complex I via ND3 subunit binding; increased AMP/ATP ratio; mild ROS elevation (1.8×); mPTP stabilization via VDAC1	AMPK activation, tumor cell metabolic stress induction, cardiomyocyte protection under chemotherapy
Liver [37]	CRTC2 inactivation; decreased PEPCK (−72%) and G6Pase (−68%) expression; gluconeogenesis inhibition	Improved insulin sensitivity and hepatic glucose output reduction
Skeletal Muscle [38]	AMPK-mediated GLUT4 translocation (+58%); increased PGC1α-driven mitochondrial biogenesis (+35–40%)	Enhanced glucose uptake and mitochondrial function, improved insulin responsiveness
Cardiomyocytes [39]	Mitochondrial membrane potential stabilization via VDAC1 binding	Reduction in doxorubicin-induced cardiotoxicity and maintenance of cardiac function
Gut Microbiome [40]	*Akkermansia muciniphila* expansion (+12-fold); FXR inhibition; TGR5 activation; SCFA modulation	Improved gut barrier integrity, systemic inflammation reduction, and anti-endotoxemic effects
Epigenetic Remodeling [41,42]	AMPK-mediated phosphorylation; chromatin opening at tumor suppressor loci (p21, p53); circRNA_1805 downregulation	Support for anti-aging and anticancer gene expression landscapes
Tumor Microenvironment [43,44,45]	Lactate reduction (−65%); HIF-1α suppression (−58%); increased MHC-I expression; M2-to-M1 macrophage repolarization; enhanced dendritic cell maturation and T-cell infiltration	Conversion of immunosuppressive milieu to immunostimulatory microenvironment
Systemic Immunomodulation [15,46]	NRF2 activation; SASP suppression; NLRP3 inflammasome inhibition; enhanced antiviral T-cell response	Delay in immunosenescence, reduced inflammaging, improved immune surveillance and vaccine efficacy

AMPK—AMP-Activated Protein Kinase; ATP—Adenosine Triphosphate; CRTC2—CREB-Regulated Transcription Coactivator 2; PEPCK—Phosphoenolpyruvate Carboxykinase; G6Pase—Glucose-6-Phosphatase; GLUT4—Glucose Transporter Type 4; PGC1α—Peroxisome Proliferator-Activated Receptor Gamma Coactivator 1-alpha; VDAC1—Voltage-Dependent Anion Channel 1; mPTP—Mitochondrial Permeability Transition Pore; FXR—Farnesoid X Receptor; TGR5—Takeda G-protein–coupled Receptor 5; SCFA—Short-Chain Fatty Acids; circRNA—Circular RNA; HIF-1α—Hypoxia-Inducible Factor 1-alpha; MHC-I—Major Histocompatibility Complex Class I; NRF2—Nuclear Factor Erythroid 2–Related Factor 2; SASP—Senescence-Associated Secretory Phenotype; NLRP3—NOD-like receptor family pyrin domain-containing 3.

**Table 2:** Summary of key clinical trials investigating the effects of metformin in cancer and aging.

Trial Name	Year	Population	Intervention	Primary Outcomes	Remarks
MANSMED Trial [119]	2021	124 men with high-risk prostate cancer	Metformin + ADT vs. ADT	Increased CRPC-free survival; benefit observed in localized and low-volume metastatic disease	Improved disease control; no OS benefit
Metformin in Metastatic Breast Cancer [120]	2019	40 non-diabetic ER/PR+ metastatic breast cancer patients	Metformin + chemotherapy vs. placebo	No difference in response rate, PFS, or OS	No survival benefit despite tolerability
Neoadjuvant Metformin Breast Cancer Trial [121]	2022	80 non-diabetic women with locally advanced breast cancer	NACT + metformin vs. NACT	Increased ORR, cCR, pCR, and BCR; serum metformin positively correlated with outcomes	Enhanced clinical/pathological response
MA.32 Trial [122]	2023	3649 non-diabetic high-risk breast cancer patients	Metformin 850 mg BID vs. placebo	No reduction in invasive disease-free survival or cancer incidence	Ineffective for cancer prevention
MetBreCS [123]	2025	Postmenopausal breast cancer survivors (BMI > 25)	Metformin vs. placebo	Transcriptomic and metabolic shifts observed; decreased expression of immune genes	Potential preventive effect via tissue remodeling
CCTG MA.32 [124]	2023	2521 HR+ breast cancer patients	Metformin vs. placebo with endocrine therapy	Increased non-adherence with metformin; no difference in ET discontinuation	Emphasizes need for adherence strategies
Ovarian Cancer Phase II RCT [125]	2020	108 advanced ovarian cancer patients	Metformin + chemo vs. placebo	No improvement in PFS or OS	Safe but ineffective as adjunct
The METNEO Study [126]	2024	70 non-diabetic breast cancer patients randomized 1:1	Neoadjuvant AC-T chemotherapy (Adriamycin + Cyclophosphamide followed by Paclitaxel) with or without metformin (850 mg BID)	Improved clinical response; increased breast-conserving surgery; higher pCR; DR4/DR5 mRNA upregulation; CD133^+^ cancer stem cell reduction	Enhances neoadjuvant efficacy Metformin modulates apoptosis and stemness by upregulating death receptors and downregulating CD133^+^ stem cells, acting as a molecular chemosensitizer
Phase II RCT [127]	2025	108 patients with advanced-stage ovarian cancer (54 in each arm); majority received neoadjuvant chemotherapy (66%) or primary debulking surgery (31%); 88% had high-grade serous histology	Platinum/taxane-based chemotherapy + Metformin 850 mg orally BID (n = 54) vs. placebo (n = 54); followed by 2-year maintenance therapy (metformin or placebo)	No significant improvement in PFS or OS	Addition of metformin was safe and well tolerated but did not result in significant improvements in survival outcomes
FAZA-PET Hypoxia Trial [128]	2022	Stage IB–IVA cervical cancer with FAZA+ tumors	Metformin + chemoradiotherapy	Reduced tumor hypoxia; trend toward improved DFS	Potential radiosensitizer; improves hypoxia
DLBCL RCT [129]	2024	100 adult patients with histologically confirmed DLBCL eligible for first-line R-CHOP, PS ≤ 2, life expectancy ≥ 6 months; randomized 1:1 (50 per arm)	R-CHOP chemotherapy ± Metformin; dosage unspecified in abstract	Higher CR; lower relapse/progression; reduced mortality; improved DFS, PFS, and OS	Metformin addition significantly improved remission, reduced relapse and mortality, and extended DFS, PFS, and OS in DLBCL patient
TAXOMET Trial [130]	2019	99 mCRPC patients	Docetaxel + metformin vs. placebo	No significant difference in PSA response, PFS, or OS	Limited synergy with docetaxel
TAME Trial [131]	Ongoing	3000 non-diabetic older adults aged 65–79 years, free from major chronic illness at baseline; multi-center, placebo-controlled trial planned across U.S. aging research centers	Metformin 1500 mg/day vs. placebo for up to 4 years	Designed to detect reduction in incidence of age-related chronic diseases (e.g., MI, stroke, cancer, dementia, mortality)	TAME is the first large-scale randomized trial to test whether a generic drug can delay multiple age-related diseases simultaneously; outcome may redefine aging as a modifiable risk factor
MILES Trial [132]	2018	Older adults (mean age ~70 years), non-diabetic, relatively healthy, recruited for short-term mechanistic aging study; designed as a randomized, placebo-controlled trial	Metformin 1500–2000 mg/day vs. placebo for 6 weeks	Significant changes in aging-related gene expression: increased mitochondrial function and decreased inflammatory signaling	Short-term metformin treatment showed transcriptional rejuvenation, including mitochondrial biogenesis, decreased inflammatory signaling, and metabolic reprogramming; supports metformin’s role in modulating key aging pathways at a molecular level

ADT—Androgen Deprivation Therapy; AC-T—Adriamycin (doxorubicin), Cyclophosphamide, followed by Taxane; BID—Twice Daily; BMI—Body Mass Index; cCR—Clinical Complete Response; CR—Complete Response; CRPC—Castration-Resistant Prostate Cancer; DFS—Disease-Free Survival; DLBCL—Diffuse Large B-cell Lymphoma; DR4/DR5—Death Receptor 4/5; ET—Endocrine Therapy; FAZA—Fluoroazomycin Arabinoside; HR—Hazard Ratio/Hormone Receptor; mCRPC—Metastatic Castration-Resistant Prostate Cancer; mo—Months; NACT—Neoadjuvant Chemotherapy; ORR—Overall Response Rate; OS—Overall Survival; pCR—Pathological Complete Response; PFS—Progression-Free Survival; PR—Partial Response; RCT—Randomized Controlled Trial; R-CHOP—Rituximab, Cyclophosphamide, Doxorubicin, Vincristine, and Prednisone.

## Data Availability

All data arising from this study are included within the article.

## Abbreviations

The following abbreviations are used in this manuscript:

**Abbreviation**	**Full Form**
*A. muciniphila*	*Akkermansia muciniphila*
AC-T	Adriamycin and Cyclophosphamide followed by Taxane
AKT	Protein Kinase B
AMPK	AMP-activated Protein Kinase
AMP/ATP	Adenosine Monophosphate/Adenosine Triphosphate
BID	Twice Daily
BMI	Body Mass Index
BCS	Breast-Conserving Surgery
CAR-T	Chimeric Antigen Receptor T-cell
CD107a	Cluster of Differentiation 107a
circRNA	Circular RNA
CR	Complete Response
CRPC	Castration-Resistant Prostate Cancer
CRTC2	CREB-Regulated Transcription Coactivator 2
CTL	Cytotoxic T Lymphocyte
CTLA4	Cytotoxic T-Lymphocyte-Associated Protein 4
DC	Dendritic Cell
DFS	Disease-Free Survival
DLBCL	Diffuse Large B-cell Lymphoma
DR4/DR5	Death Receptor 4/5
ERAD	Endoplasmic Reticulum-Associated Degradation
ET	Endocrine Therapy
FAZA	Fluoroazomycin Arabinoside
FHV	FAZA Hypoxic Volume
FOXP3	Forkhead Box Protein P3
FAS	Fatty Acid Synthase
FXR	Farnesoid X Receptor
G6Pase	Glucose-6-Phosphatase
GI	Gastrointestinal
GLUT4	Glucose Transporter Type 4
H2BS36ph	Histone H2B Serine 36 Phosphorylation
H3K27me3	Tri-methylation of Lysine 27 on Histone H3
HER2	Human Epidermal Growth Factor Receptor 2
HIF-1α	Hypoxia-Inducible Factor 1-alpha
HR	Hazard Ratio/Hormone Receptor
IFN-γ	Interferon-gamma
IGF1	Insulin-like Growth Factor 1
IL-6	Interleukin-6
IRS-1/2	Insulin Receptor Substrate 1/2
LVEF	Left Ventricular Ejection Fraction
MA.32	Metformin Atrial Trial 32
MCI	Mild Cognitive Impairment
MDSC	Myeloid-Derived Suppressor Cells
MET	Metformin (as nanoparticles)
MQ	Macrophage
mCRPC	Metastatic Castration-Resistant Prostate Cancer
MHC-I	Major Histocompatibility Complex Class I
mPTP	Mitochondrial Permeability Transition Pore
mTOR	Mammalian Target of Rapamycin
mTORC1	mTOR Complex 1
NACT	Neoadjuvant Chemotherapy
NF-κB	Nuclear Factor kappa-light-chain-enhancer of activated B cells
NK cell	Natural Killer Cell
NLRC4	NLR Family CARD Domain-Containing Protein 4
NLRP3	NOD-like Receptor Family Pyrin Domain-Containing Protein 3
NRF2	Nuclear Factor Erythroid 2–Related Factor 2
NR	Not Reached
NSCLC	Non-Small Cell Lung Cancer
ORR	Overall Response Rate
OS	Overall Survival
P-AKT	Phosphorylated AKT
P-AMPK	Phosphorylated AMPK
P-NLRC4	Phosphorylated NLRC4
P-PD-L1 Ser195	Phosphorylated Programmed Death-Ligand 1 at Serine 195
P-PI3K	Phosphorylated Phosphoinositide 3-Kinase
PC	Pemetrexed + Cisplatin
PEPCK	Phosphoenolpyruvate Carboxykinase
pCR	Pathological Complete Response
PGC1α	Peroxisome Proliferator-Activated Receptor Gamma Coactivator 1-alpha
PhenoAge	Phenotypic Age
PR	Partial Response
RCT	Randomized Controlled Trial
R-CHOP	Rituximab, Cyclophosphamide, Doxorubicin, Vincristine, and Prednisone
ROS	Reactive Oxygen Species
SASP	Senescence-Associated Secretory Phenotype
SCFA	Short-Chain Fatty Acids
SIRT3	Sirtuin 3
T-DM1	Trastuzumab Emtansine
TAME	Targeting Aging with Metformin
Tcm	Central Memory T Cell
Tem	Effector Memory T Cell
TET2	Ten-Eleven Translocation Methylcytosine Dioxygenase 2
TIM-3	T-cell immunoglobulin and mucin-domain-containing-3
TGR5	Takeda G-protein–coupled Receptor 5
TME	Tumor Microenvironment
Treg	Regulatory T Cell
ULK1	Unc-51 Like Autophagy Activating Kinase 1
VEGF	Vascular Endothelial Growth Factor
VDAC1	Voltage-Dependent Anion Channel 1
